# Patient Experience in Pancreas-Kidney Transplantation—A Methodological Approach Towards Innovation in an Established Program

**DOI:** 10.3389/ti.2022.10223

**Published:** 2022-04-14

**Authors:** Pedro Ventura-Aguiar, Beatriu Bayés-Genís, Antonio J. Amor, Miriam Cuatrecasas, Fritz Diekmann, Enric Esmatjes, Joana Ferrer-Fàbrega, Ángeles García-Criado, Mireia Musquera, Silvia Olivella, Eva Palou, David Paredes, Sonia Perea, Anna Perez, Esteban Poch, Barbara Romano, Joan Escarrabill

**Affiliations:** ^1^ Nephrology and Kidney Transplant Department, Hospital Clinic Barcelona, Barcelona, Spain; ^2^ Laboratori Experimental de Nefrologia i Trasplantament, Fundació Clínic, August Pi i Sunyer Biomedical Research Institute (IDIBAPS), Barcelona, Spain; ^3^ Endocrinology Department, Hospital Clinic Barcelona, Barcelona, Spain; ^4^ Pathology Department, Center for Biomedical Diagnosis, Hospital Clinic Barcelona, Barcelona, Spain; ^5^ Red de Investigación Renal (REDINREN), Madrid, Spain; ^6^ Hepato-Bilio-Pancreatic Surgery and Digestive Transplant Unit, Hospital Clinic Barcelona, Barcelona, Spain; ^7^ Radiology Department, Center for Imaging Diagnosis, Hospital Clinic Barcelona, Barcelona, Spain; ^8^ Urology Department, Hospital Clinic Barcelona, Barcelona, Spain; ^9^ Patient Experience, Hospital Clinic Barcelona, Barcelona, Spain; ^10^ Transplant Coordination Department, Hospital Clinic Barcelona, Barcelona, Spain

**Keywords:** diabetes mellitus, simultaneous pancreas-kidney transplantation, chronic kidney failure, patient care, organizational innovation, focus groups

## Abstract

Simultaneous pancreas-kidney transplantation (SPKT) leads to increased survival and quality of life, and is an alternative treatment for insulin-dependent diabetes mellitus and end-stage kidney disease. Due to the particularities of this population (often with multiple comorbidities) and of the surgery (only performed in a few centers), a comprehensive analysis of patients’ experience along the SPKT process is crucial to improve patient care and add value to this procedure. Therefore, we applied a systematic and iterative methodology with the participation of both patients and professional teams working together to explore and identify unmet needs and value-adding steps along the transplant patient journey at an established pancreas transplant program. Four main steps (to comprehend, to explore, to experiment and to assess) led to several interventions around three major areas: Administration and logistics, information and communication, and perceived quality of assistance. As a result, both displacements to the hospital for diagnostic purposes and the time delay involved in joining the patient waiting list for transplantation were reduced in parallel to the administrative procedures. In conclusion, the methodological implementation of key organizational changes has great impact on overall patient experience. Further quantitative analysis from the patient’s perspective will consolidate our program and may add new prototype service design components.

## Graphical Abstract

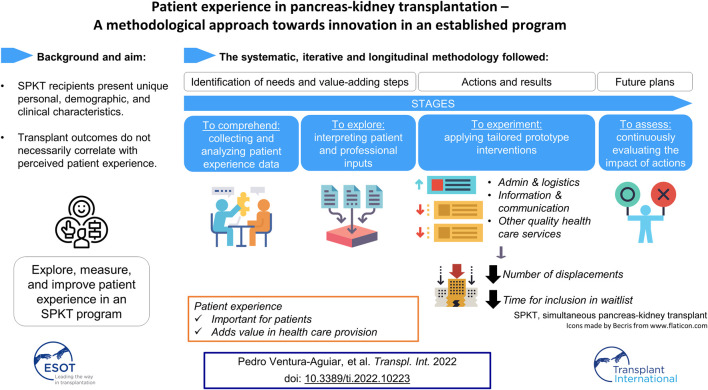

## Introduction

In Type 1 diabetes mellitus (T1DM), the immune destruction of pancreatic beta cells leads to deficient production of insulin and renders patients dependent on life-long exogenous insulin therapy. Approximately 50% of diabetic patients develop serious complications, including chronic kidney disease (1), which was responsible for approximately 82,000 deaths worldwide and 3 million disability-adjusted life years (DALYs) in 2019 (2). Diabetic nephropathy is the leading cause of end-stage renal disease (ESRD) (3, 4). In these cases, simultaneous pancreas and kidney transplant (SPKT) is preferred over kidney transplant alone as it leads to increased patient and kidney graft survival rates (5–7). Moreover, since SPKT restores both organ functions in a single procedure, it overcomes the need for dialysis, insulin therapy, dietary restrictions and, most importantly, it minimizes diabetic complications (8, 9).

Concomitant improvement in quality of life (QoL) and other patient-reported outcome measures (PROMs) have also been extensively reported in cross-sectional studies including SPKT patients (10–14). However, none included patient reported experience measures (PREMs) throughout the transplant process. In this regard, several authors agree that prioritizing what patients value is key in quality healthcare provision. In the last years, patient’s appraisal of their own experience with healthcare services has received much attention, with an ever-increasing number of studies that consider it in the design and upgrade of health systems (15–18). The major challenge lies in translating the heterogeneity of individual patient experience into measurables categories. For this, identifying the stakeholders involved in patient care and defining the patient journey map are useful to sort and characterize the added-value and non-added-value steps in the healthcare process (19). Qualitative data can subsequently be collected by methods such as interviews with patients, surveys and focus groups (19–22).

Herein we present a study aimed at integrating patient experience into qualitative healthcare assessment within the Pancreas-Kidney Transplant Program at the Hospital Clinic Barcelona (HCB). In order to achieve this, we followed a systematic, iterative and longitudinal research methodology to acquire data from patients and professionals while they interacted with each other.

## Patients and Methods

### Study Design

**FIGURE 1:**
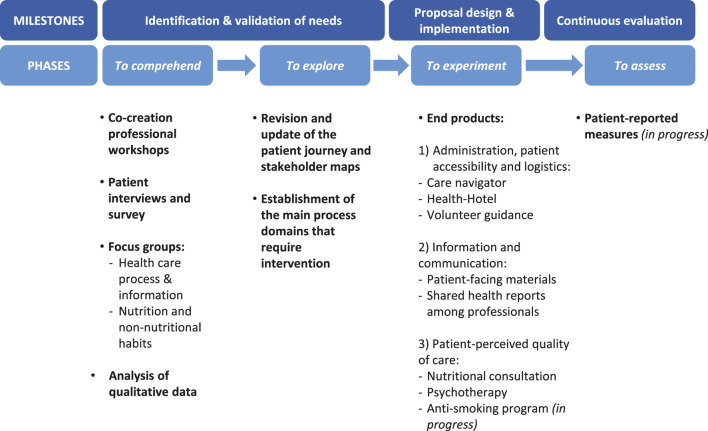
In our project journey towards the improvement of the healthcare service delivered by the HCB’s transplant unit, professionals and patients interacted with one another to provide feedback while engaging in different activities. These were organized in four phases to identify and confirm current unmet needs of our healthcare system (to comprehend and to explore) and to put solutions into practice (to experiment). Those proposals that have already been implemented are currently under assessment.

We designed a systematic methodology to assess patient experience and improve the quality of the well-established Pancreas-Kidney Transplant Program at the HCB. This patient-centered project was developed in four phases that aimed to identify and validate current unmet needs and/or value-adding steps in our transplant process of care, as well as implementing specifically designed prototype proposals (Figure 1):

1) To comprehend—to collect and analyze data regarding the status of patient experience at the HCB (Pancreas-Kidney Transplant Program) from both professional and patient sources. Specifically, a team of professionals revised the relevant literature and were brought together at five co-creation workshops. The activities involved mixed patient-professional teams taking part in three focus groups, a patient interview, an online survey and several open informative events for patients on social media (23–26).
2) To explore—to dissect and interpret the newly acquired information on uncovered or upgradable healthcare domains while checking to what extent they can be generalized.
3) To experiment—to design and implement new proposals according to the unveiled unmet needs.
4) To assess\to continuously evaluate the impact of the novel processes applying PREMs (currently in progress).

All these steps were carried out at the HCB, Spain, between October 2020 and February 2021. HCB performs an average of 20 pancreas transplants per year and is the main referral hospital for patients from five Spanish autonomous regions as well as Andorra (27, 28).

### Study Participants

#### Healthcare Professionals

In 2019, the HCB established the Patient Experience Team, which is a living lab and multidisciplinary group of professionals (a sociologist, psychologist and physician) who work on the evaluation of the patient experience and on the design and analysis of PREMs following implementation of new protocols (29–31).

For this study, a total of 13 healthcare professionals from different disciplines and educational backgrounds were involved, including members from the HCB Patient Experience Team and others (physicians, nurses, administrative staff, a nutritionist and a participatory health care consultant). Professionals were involved in all co-creation workshops and focus groups.

#### Patients

A total of 12 patients worked together with the multidisciplinary professional team. Five patients participated in the focus group sessions, five responded to a logistics survey by email and two were interviewed online on World Diabetes Day 2020 (23). Patient selection was made according to clinical and demographic data and aimed to represent all patient archetypes that had been defined during the previous co-creation workshops.

### Data Collection and Analysis

#### Focus Groups

Focus groups were carried out virtually and lasted between 60 and 90 min. Prior to the sessions, the focus group agenda was agreed on by the multidisciplinary professional team. Following contact, patients willing to participate received detailed information regarding the objective of the session, connection instructions as well as the consent form to participate and be recorded. Focus group sessions were moderated by two members from the patient experience team. Of relevance, principal care physicians did not participate in these sessions, to avoid biasing patients’ responses and interaction. In each session, the moderators introduced the purpose and aims of the study. Participants were also reminded that they would be recorded, and that all data collected during the session would be treated anonymously and confidentially. At the end of the session, patients were asked an open-end question in order to gather further feedback and/or suggestions.

**FIGURE 2:**
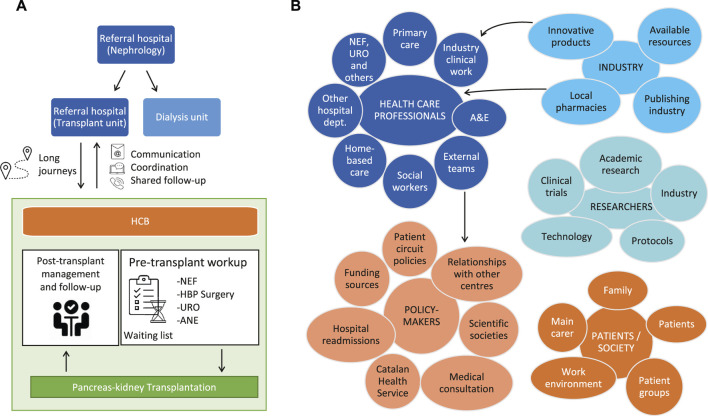
**(A)** Patient journey and **(B)** stakeholder mapping helped to spot several pain points for patients undergoing a double pancreas-kidney transplant. ANE, Anesthesiology; A&E, Accident and Emergency; HCB, Hospital Clinic Barcelona; HBP, Hepatobiliopancreatic surgery; NEF, Nephrology; URO, Urology.

During the first focus group, patients validated the general areas of improvement identified during the previous process mapping (Table 1; Figure 2), patient interview and survey (Supplementary Tables S1, S2). Afterwards, a formal script was prepared (Supplementary Table S3) for the second and main focus group about the healthcare process & information. Here, patients helped to identify the specific domains that needed to be addressed in the transplant unit and discussed them extensively (Table 2). The session was also useful for gaining aware of the emotions that were generated in each step of the care process (Figure 2).

**TABLE 1:** Pre-identified areas of interest for transplant patients according to professional opinion.

Key moments during the SPKT process	Areas of interest
At the time of referral to the HCB	The healthcare process that takes place at the HCB. This information must be given to the referral center.
	General information provided to each patient through HCB’s Portal Clínic platform (42), QR code, etc.
	The details of the contact person before the first visit to the HCB.
	Information that should be provided by the patient: Medical report from their center of origin, diagnostic digital images.
	Legal information (especially relevant to foreigners).
	Access information for the first visit at the HCB.
	Available public services around the HCB such as the patient hotel.
During the candidate assessment for SPKT	Information to be given to the patient during the first visit to the HCB: All kinds of involved health professionals, the place, number and types of visits prior to the SPKT and the complementary and exploratory analyses.
	The duration of the assessment process.
	Overall information on the SPKT.
	Criteria for medical decisions.
	Contraindications of the SPKT (obesity, etc.).
	Patients at risk: Nutrition, smoking habit, alcohol, addictions, etc.
	Social acceptance.
During the waiting time and at the time of transplant surgery	Time management until the surgery date. Important topics to be addressed: Prioritization criteria and possible unexpected complications during the assessment and waiting period, given that they are fragile patients.
	Follow-up during the waiting period (analyses and periodic explorations) and contact channel for possible clinical incidents.
	Removal of the donor organ and viability assessment: Safety criteria and risk of donor incompatibility at the last moment (50% of patients cannot receive the organ after the first call).
	Informed consent before acceptance onto the patient waiting list for transplantation.
	Events that take place the day of the call (immediately getting to the HCB) and analyses that need to be carried out and/or repeated.
	Information for the caregiver.
At hospital discharge and follow-up	Pharmacological treatment: Lifelong prescriptions, adherence and secondary effects (vision, blood pressure, skin, tremor, etc.).
	Changes in nutritional habits (such as increased appetite) and food safety.
	Everyday life: Travelling, pets, vaccinations, and sexual and physical activity.
	The importance of smoking cessation.
	Follow-up information during outpatient care: First quarter, first year and thereafter.
	Benefits of shared follow-up with doctors and nurses and how this will take place.
	Contact details (email and phone).
	Warning signs and symptoms (infection and rejection).
	Asymptomatic hypoglycemia.
	Maintaining diabetes under control and possible complications (endocrinologic, cardiac, ophthalmologic, etc.).

HCB, Hospital Clinic Barcelona; SPKT, simultaneous pancreas-kidney transplant.

**TABLE 2:** Collected data during the focus group session on information and healthcare assistance.

Meta-category	Category	Results	Selected patient quotations
Contact and Communication	Contact via telephone	Most of the patients do not require any phone calls for urgent issues. Nevertheless, if that happened, they would like quick and effective telephone access.	*I think that, if I were involved in an urgent situation, I would try to call the hospital.*
	Contact via email	It was highly rated by those who used it, although they would appreciate a quicker reply (<48 h).	*I send emails to the Unit now and then when I have doubts. They don’t reply immediately, they take a couple of days, but they usually reply*.
	Displacements to the hospital (pre- and post-transplant)	The pre-transplant phase does not require many displacements. After the procedure, they go through check-ups every 4–5 months, which usually require less than a day. Also, due to the COVID-19 pandemic, patients try to avoid public transportation.	*I have scheduled visits every 6 months or so with the endocrinologist at the HCB, but I see my private ophthalmologist and the rest of the specialties here in San Sebastián*.
	Communication between hospital of origin and HCB	Inter-hospital miscommunication was mentioned and some patients experienced issues with the transfer of their files. This caused longer waiting times and more anxiety. A shared digital platform for medical histories was suggested to ease data access and increase health system efficiency.	*My endocrinologist recommended medical consultation with the HCB for this type of intervention. I underwent several tests for 2 years and when my file was ready to be transferred, it got lost and had to be redone. It was such a long process […]*.
Information	Previous information	Most patients agreed that the information they had received about the SPKT was clear and honest but probably not enough, especially for highly vulnerable patients.	*I mean receiving more information such as what a double transplant is, etc. […] As you can well imagine, when they tell you that [the need for a double organ transplant] you have no other choice than to adapt and make plans for a new life. In my case, I needed much more information…*.
	Information on waiting time	There is room for improvement here too. Patients would like to have more knowledge of the waiting time. Even rough estimates would be useful to be psychologically more prepared and better organize their everyday lives.	*I didn’t feel anxious while waiting, but I would have preferred a bit more extra time to conclude some matters or to better plan them. For example, the week before the transplant I bought a car and right before getting to the HCB I had to deal with some paperwork. If I had known a month in advance about the possibility of an imminent organ donor, I would have postponed my purchase. You have your own life and events continue to unfold, but the moment you receive the call you’re certain that it will all change […]*.
Waiting time	Waiting time	There was a great variety of opinions. Those who had added health complications or came from far away recalled a long wait.	*In my case, I received the first transplant very quickly, but then I rejected it and had to wait over 5 years for the second one.*
	Consequences of waiting time	The majority of patients were convinced that longer waiting times have physical consequences. Some of them have experienced it. As a result, they stressed the importance of receiving the new organs as soon as possible.	*People tend to associate diabetes with a different lifestyle, but they forget about all the problems that may suddenly arise. In my case, one of my feet burst, my vision got worse and I don’t know what else I could have had. Maybe, if the waiting time had been shorter, we would have avoided or minimized such events. On the other hand, I understand that other surgeries are going on at the same time…*.
Impact on patients’ everyday life	Family and social awareness	Having a serious illness and going through such a delicate procedure helps increase awareness.	*I have experienced it in my family too. They now see organ transplantation very differently. My friends from the swimming club now give blood. People are more conscious if they know of someone who is going through that*.
	Improvements in working life	SPKT improves patients’ professional life too. They were able to work afterwards.	*I started working for ONCE as a lottery ticket seller. I became blind in 2008, I started dialysis in 2010, I was transplanted in 2013 and then, 4 years later, I found this job. I am entitled to a disability pension, but I can work and honestly, this makes a tremendous difference*.
	Transplants that are finally not performed	The fact that sometimes pre-scheduled transplants cannot be performed cause a great deal of distress to patients. Still, they are sympathetic towards medical decisions.	*This is hard. I had reached an impasse right before the second transplant, but I was on the reserve list and nonetheless I had to go home. “We will call you back,” they say. Another year…*.
	Psychological support	Patients agreed to receiving emotional support, especially during (but not limited to) the waiting time and after the transplant in order to adjust to new living and working conditions. Psychological aid may be appropriate.	*I finally relaxed, but you pay for all the stress that you have suffered during the previous months. Then I was alone, and it took me a while before I realized I was depressed*.
	Improvements in everyday life	Everyone agreed that there is a substantial improvement in their daily life after the transplant.	*You feel so much better after the transplant. The rest of your activities improve. The freedom you get to move around is of great importance to me*.

HCB, Hospital Clinic Barcelona; ONCE, Spanish National Organization for the Blind; SPKT, simultaneous pancreas-kidney transplant.

MAXQDA software (VERBI GmbH, Germany) (32) was used to analyze the data from the verbatim transcriptions of the recorded focus group sessions. The analyses gave rise to the coding of meaning units (all expressions that have the same meaning) which were then combined into meta-categories. Further qualitative analyses (absolute frequency of meaning units) were performed according to the COREQ criteria for qualitative research (33, 34).

#### Patient Data

Patient data regarding the variables study time and number of displacements were collected from patients’ electronic registries from 2019 to June 2021. Study time was defined as the total time since the first evaluation for pancreas transplantation until clinical decision regarding inclusion/exclusion of the patient in/from the waiting list. The number of displacements were defined by the number of visits to the HCB during the pre-transplant workup. Mean and standard deviation (SD) were used for these quantitative continuous data.

## Results

The methodology applied in this study led to the identification of key points and unmet needs as well as the implementation of novel protocols and circuits. To highlight the relevance of this stepwise systematic approach, the results obtained in each step will be described separately.

### To Comprehend—Understanding Patient Experience

#### The Professional Viewpoint: Co-Creation Workshops and Literature Review

During the co-creation workshops, patient archetyping, stakeholder and patient journey mapping and categorization of the transplant process were carried out by professionals to identify potential key steps for patients undergoing a double pancreas-kidney transplant.

Based on the literature review and professional experience, professionals classified pancreas transplant candidates into a number of archetypes, according to age (<45 or >45=years), residence zone (Barcelona, Catalonia or other autonomous regions), social and family support (good or dependent), Body Mass Index (BMI) (BMI > 27: High or BMI < 20: Low), vascular complications (micro or micro and macrovascular) and type of DM (T1DM or T2DM). Patient archetypes were used to select focus group participants to assure representation of all archetypes during the sessions.

The major stakeholders in our healthcare system were mapped as: Professionals from different medical specialties, from other disciplines and from public and private research and industry; policy-makers and society at large (including patients and caregivers). While defining the patient journey, three main dynamics were taken into consideration. Firstly, referral from multiple centers implies an administrative burden. Secondly, there is a high number of patients travelling long distances from other cities within the same region (30%) or from other autonomous regions (40%–50%). Finally, the pre-transplant workup before a clinical decision regarding inclusion in/exclusion from the patient waiting list for transplantation is a complex procedure (Figure 2).

Professionals further characterized the pancreas transplant process into four steps which were of potential interest for intervention. These were defined as: 1) Referral to pancreas transplantation, 2) workup and candidate assessment for SPKT, 3) wait listing and transplant day, and 4) hospital discharge and follow-up (Table 1).

#### The Patient Viewpoint: Individual Interviews and Survey

To explore individual patients’ perspectives, a live online interview with two pancreas-kidney transplant recipients was broadcasted on World Diabetes Day (23). During this interview (Supplementary Table S1), questions were raised concerning five relevant areas: Challenges in everyday life (work, education, leisure and others), treatment (management, compliance, medical check-ups, complications and hospitalizations, adverse events, etc.), required information (pre- and post-transplant), emotional impact (due to the physical change after the transplant, anxiety, fear, feeling of insecurity, etc.) and overall impact on the family and social environment. Data from the interviewees as well as comments and questions raised by the audience were recorded for further analysis.

Additionally, five patients responded to a survey on logistics requirements for patients coming from other regions during the COVID-19 pandemic. According to them, the areas that needed improvement were the limited visiting hours and comfort currently offered by the hospital as well as other affordable alternatives to lengthy daily travelling. Patients’ response to the survey questions and their suggestions for improvement are shown in Supplementary Table S2.

#### The Patient Viewpoint: Focus Groups

**FIGURE 3:**
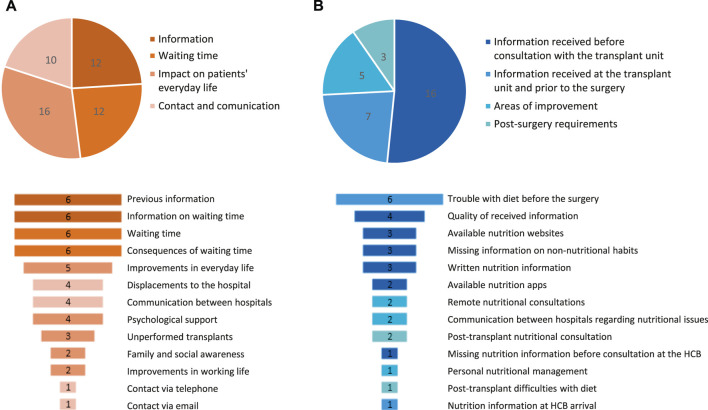
Meta-categories (upper graphs) and categories (lower graphs) of patient preferences and needs that were identified during the focus group sessions: **(A)** Healthcare process & information (*N* = 50 preferences/needs) and **(B)** nutrition and non-nutritional habits (*N* = 31 preferences/needs). Absolute frequencies are shown. HCB, Hospital Clinic Barcelona.

The most important categories reported by patients during the focus groups were receiving sufficient information prior to the intervention and the waiting time for transplantation and its consequences (Figure 3A). The latter may correspond to patients at the most severe clinical stage and for whom transplantation could imply more serious complications. Patients also highlighted the importance of having rough estimates for the transplantation date to better organize their personal and work life and to decrease anxiety during this period (Table 2).

In addition to this, the emotional impact caused by the SPKT was also discussed. Although they all agreed that their quality of life had improved, emotional support would have been appreciated too, for instance in terms of psychological follow-up (Figure 3A). This was especially relevant during the adaptation process after the transplant and throughout the waiting period. The need to better manage the distress caused by last-minute cancellation of their surgery was also highlighted (Table 2).

Other concerns raised by patients were those related to their displacements to and from the HCB and to communication between the hospitals, especially for patients that had been treated in more than one center (Figure 3A; Table 2). According to patients, administrative barriers such as the delayed transfer of medical records between hospitals usually increase the waiting period and trigger anxiety. A full description of focus group results is given in Table 2.

### To Explore—Interpreting Patient and Professional Input

Following the input obtained from the interviews, survey and focus group sessions, the patient journey and stakeholder maps were reviewed and updated (Figure 2).

The analysis of qualitative data from the main focus group yielded 50 unmet needs, which were grouped into 13 categories and 4 meta-categories: The information received throughout the process; the waiting time; the impact of the SPKT on patients’ day-to-day life; and the contact and communication with the HCB before, during and after the transplant (Figure 3A). Finally, the third nutrition-oriented focus group (Supplementary Table S4) spotted 31 categories that were grouped into 4 meta-categories. The main ones were those related to the amount and quality of nutrition information received before the intervention, especially regarding fluid intake restrictions (Figure 3B; Table 3).

**TABLE 3:** Collected data during the focus group session on nutrition and other non-nutritional habits.

Meta-category	Category	Results	Selected patient quotations
Information received before consultation with the transplant unit	Missing nutrition information before consultation at the HCB	Only a minority reported not having received any kind of nutritional guidance before contacting the HCB.	*I was unlucky with this [nutritional consultation]. My doctor retired around the time they called me regarding the transplant. I didn’t have any nutritionist during the first transplant either*.
	Written nutrition information	Patients confirmed they had received such information on paper.	*I was given plenty of written dietary information such as home recipes and books. I had already decreased the amount of salt and given up smoking*.
	Available nutrition apps	Some patients received the names of apps to help them design appropriate dietary patterns.	*They encouraged us to download an app with preestablished meals and cooking tips during the time I was on dialysis, to make it easier to bear*.
	Available nutrition websites	Internet was also an option for some of them to find dietary patterns which, in most cases, led to successful search results.	*I had access to the internet and could get information on the protein and potassium content of certain foods. I also checked different activities that I could do. I felt this was necessary*.
	Missing information on non-nutritional habits.	Despite available nutritional guidance, they had not been informed about other healthy habits like exercising and quitting smoking. However, they were already aware and tried to follow them.	*I wasn’t told but I’ve always exercised and never smoked. That was a personal choice. I used to go to the gym, cycle, run, etc., even looking after the elderly, everything I could physically do except swimming to avoid infection of the peritoneal tubes*.
	Quality of received information	In general, nutritional recommendations before arriving at the HCB were considered adequate.	*At the Hospital Complex of Navarre, we had nutrition services that I received at the pre- and post-transplant stages and during dialysis while working together with the nurses. I also saw a personal nutritionist through the Renal Disease Association for a year and a half*.
Information received at the transplant unit and prior to the surgery	Nutrition information at HCB arrival	Some patients did not receive further instructions or recommendations as they already had them in abundance.	*Not in my case. Apart from the visits with my regular doctor, I didn’t have any with nutrition specialists. I may have got some advice, but it was minor. Lately, I’ve visited the endocrinologist, but only a couple of times*.
	The trouble with diet before the surgery	This was one of the most popular and anxiety-inducing topics. There was unanimity among patients on fluid intake (and not food) as the most troublesome dietary issue before the transplant.	*Water becomes an obsession. When I had to be treated intravenously, I remained obsessed with liquids 24/7. The drinking situation is overwhelming*.
Areas of improvement	Personal nutritional management	Overall healthcare assistance could be improved if personal and individualized nutrition therapy was offered.	*I think that we need more nutrition treatment, and this should be more personalized. I received a lot of information about diets. However, I miss having professional support, someone to talk to and who follows up on you*.
	Remote nutritional consultations	Telemedicine could be applied, whenever possible, for those who live far away from the HCB.	*Regardless of your location, I reckon that videoconferences are a good communication channel*.
	Communication between hospitals regarding nutritional issues	Patients agreed that this should be improved towards a shared information system.	*In my experience, the nephrologist I was seeing in Alicante was not communicating with the HCB. Once, when I was having an organ rejection, I had to drive by myself to the A&E service in Barcelona even though my blood sugar was already at 600*.
Post-surgery requirements	Post-transplant difficulties with diet.	Although patients have some diet restrictions, it is not a major problem for them.	*After the second transplant, I was told I could eat normally, although all this food contained sugar, even fruit. Sugar in excess is not good for a non-transplant person either and it forces the pancreas (which is not yours) to release insulin*.
	Post-transplant nutritional consultation	This is not a major concern either since they usually have enough information on dietary patterns to follow.	*In my case, I don’t require any nutritionist support anymore because I’ve been a diabetic person all my life and I’m more than used to dietary restrictions. To be honest, I’ve never changed my food habits except when I was on dialysis and had to watch the levels of potassium and phosphorus. I’ve always been in good shape and fit too*.

A&E, Accident and Emergency; HCB, Hospital Clinic Barcelona.

To sum up, these results led to the understanding that there were three major domains encompassing the main meta-categories identified (Figure 3): 1) Administration, patient accessibility and logistics; 2) patient-facing information and shared health reports between professionals and 3) patient-perceived quality of care throughout the transplant process regarding emotional impact, nutritional support and other non-nutritional habits (Figure 1).

### To Experiment—Designing and Applying Tailored Prototype Proposals

Following the establishment of the main domains requiring interventions to improve patient experience, a set of protocols and proposals were co-designed between professionals and patients. Protocols were further categorized regarding three major considerations for their implementation, such as pertinence, opportunity and available resources.

From an administrative point of view, the circuit of care was optimized by creating a new care navigator role, of which the main duties are to centralize and coordinate patient visits to the outpatient clinic to perform diagnostic and other complementary tests. In consequence, we observed that both patient eligibility assessment time and the number of displacements to the HCB before acceptance onto the patient waiting list for transplantation were reduced. During 2020 and in the first 6 months of 2021, and despite being an atypical period due to the coronavirus pandemic, the study time decreased by 29.3% and 73.3% and the number of displacements, by 19.2% and 45.2% compared to 2019, respectively (Table 4).

**TABLE 4:** Study time and number of displacements for joining the patient waiting list for transplantation.

	2019	2020	2021^a^
Study time, months			
Mean (SD)	7.5 (3.1)	5.3 (3.2)	2.0 (1.0)
Displacements to and from hospital			
Mean (SD)	7.3 (3.2)	5.9 (2.6)	4.0 (2.7)

a January to June.

SD, standard deviation.

To overcome the patient-reported unease surrounding the first hospital visit and the tight schedule of the pre-transplant workup, a transplant patient welcome protocol was introduced, which included the use of a patient hotel (Health-Hotel) and the volunteer guidance. On the one hand, the Health-Hotel was set up near the HCB as a result of a joint public-private partnership between the HCB and the hotel sector. Besides offering more comfortable stays to patients and accompanying adults, this project was intended to alleviate their travelling and/or accommodation expenses (as it implies no direct cost for them), avoid hospital admissions during diagnosis and shorten the post-discharge phase. On the other hand, volunteer guides offered useful first-hand information and personal accompaniment to medical appointments, depending on the patient’s comorbidities and/or impairments (visual, motor, etc.) (25, 26).

At the time of acceptance onto the patient waiting list for transplantation, patients often require a large amount of information on their procedure, treatment options, clinical benefits, etc. (Figure 3; Table 2). For this reason, we increased the printed and online resources available and organized informative patient workshops. For instance, educational videos on SPKT were posted online after receiving the approval of patients, medical societies and the Catalan Agency for Health Quality and Evaluation (AQuAS). The aim of this animated plain-language tool is to aid shared transplant decision-making (24). In addition, at the professional level, we established a quarterly and annual report system to share patient records between the HCB and other centers, therefore speeding up the data flow.

Finally, long and uncertain waiting periods, bureaucracy hurdles and the post-transplant adaptation period impact patient’s emotional wellbeing (Table 2). Hence, we allocated funding resources towards more affective support and closer follow-up through routine psychological visits. Additionally, other medical services were designed to improve the quality of care, namely pre- and post-transplant nutritional consultation at the unit and the medium-to-long-term implementation of an anti-smoking program.

## Discussion

We used a systematic strategy based on professional-patient interaction that translated into a package of potentially long-term interventions to improve the health system performance of the Pancreas Transplant Program of the HCB while upgrading patient experience.

SPKT improves clinical and non-clinical outcomes in eligible diabetic patients (5–7), (10–14). To further improve them, several authors have suggested that patient input is of utmost importance, but they do not specify how this can be put into practice. Usually, patient-reported outcomes measure QoL, psychological status or other domains with generic or specific questionnaires such as the 36-Item Short Form Survey (SF-36) or the Psychosocial Assessment of Candidates for Transplantation (PACT), respectively (10, 12–14,35). Recently, Gibbons et al. observed improvement of several PROMs while comparing post-transplant patients with those on the patient waiting list for transplantation as a surrogate of pre-transplant information. Their research was also based on qualitative interviews, which were used to better understand the impact of diabetes and kidney diseases and the transplant procedure on their QoL. Of note, diabetes-specific QoL had not improved after the surgery at least because of persistent diabetic complications, anxiety and self-imposed uninformed nutritional restrictions (13), which is in line with the emotional and nutritional support needs that were identified during the focus groups herein reported.

In contrast to these exploratory reports, and for the first time, we used patient experience assessment as a robust tool to co-design long-lasting improvement strategies and measure SPKT outcomes. Moreover, we added the focus group qualitative method analysis. Unlike individual interviews and questionnaires, these collective interviews rely on communication among participants to create and contrast data on how the system is perceived by the group in an interactive and dynamic way. Also, since group discussion is usually more stimulating than one-on-one interviews, it can give rise to more clues, insights and criticism (20,21,36).

Upon integration of focus data, several end products were implemented. Regarding logistics, the benefits of alternatives to conventional hospitalization have long been discussed (37). Among them, patient hotels, with the support from Home-Hospital units, are facilities that have been partially transformed to provide healthcare assistance and, therefore, alleviate the high demand for acute care hospital beds and other overcrowding-related problems such as nosocomial infections (38,39). By providing a Health-Hotel for patients being studied for the kidney-pancreas waiting list, we were able to concentrate outpatient visits and pre-transplant workup, which reduces the travel burden and its associated costs, and improves comfort during their stay.

Centralization of specialized care and minor procedures is common practice in healthcare organizations. This centralization may, nonetheless, lead to inequity of access to certain treatments and varying disease outcomes. In kidney transplantation, receiving dialysis more than 100 km away from a transplant center has been reported to reduce the likelihood of being referred for a transplant (40). On the other hand, pancreas transplantation is a procedure that is performed in a few centers nationwide, with patients’ referral from rural areas often implying long travelling time and costs. Therefore, minimizing the displacement requirements and costs is of the utmost importance to reduce inequity in healthcare access (41). This topic was also highlighted by patients during both the interview and focus group sessions. The introduction of a care navigator to schedule visits on the same or consecutive days, among other tasks, and the Health-Hotel protocol led to considerable savings in time and money. Conversely, the busy outpatient visits and pre-transplant workup schedule might increase patients’ already reported anxiety associated with the first contact with the Hospital. In this sense, the supporting role of HCB’s volunteers will hopefully translate into a reduction of patient uneasiness.

We prioritized actions based on their prompt implementation, which depended on readily available resources, coordination of identified gaps among hospital services and/or the need to previously shape certain professional skills and competencies. Other identified needs were not deployed immediately due to a lack of resources. Nonetheless, this methodology enabled them to be flagged as patients’ priorities and therefore they warrant adequate response in the near future.

Our work has some limitations. First, the results presented here are limited to the patient cohort, which has disease-specific requirements and several particular constraints imposed by the hospital logistics. Hence, end solutions cannot be directly extrapolated to other hospital environments without the corresponding customized variations. Secondly, the highly specific patient archetyping led to a rather small sample size. Finally, the prototype proposals are still subject to patient-based auditing to fine-tune them and hence ensure their continuity. New ones may be also designed based on the present report. In this regard, we envision future challenges such as persistent professional and patient engagement and adaptation to new protocols despite being time- and effort-consuming tasks. Furthermore, the sustained provision of organizational structures and funding will be necessary to support these interventions within a resistant healthcare culture.

In conclusion, we have shown that value in healthcare provision is ultimately revealed by taking action to improve it. In this sense, our action plan was concentrated around the areas of administration, patient accessibility and logistics (care navigator role, Health-Hotel and volunteer guidance), information and communication (patient-facing materials and shared health reports) and patient-perceived quality of assistance (nutritionist and psychologist) with promising preliminary outcomes regarding a reduced number of displacements to the hospital and reduced delay before joining the patient waiting list for transplantation. Our work also highlights the use of focus groups as a well-suited methodology to work with and for patients towards a better care system, fostering similar initiatives in other hospital units and centers.

## Data Availability

The raw data supporting the conclusion of this article will be made available by the authors, without undue reservation.

## Abbreviations

A&E, Accident and Emergency; ANE, Anesthesiology; AQuAS, Catalan Agency for Health Quality and Evaluation; BMI, Body Mass Index; DALYs, disability-adjusted life years; DM, diabetes mellitus; ESRD, end-stage renal disease; HBP, Hepatobiliopancreatic surgery; HCB, Hospital Clinic Barcelona; NEF, Nephrology; ONCE, Spanish National Organization for the Blind; PREM, patient-reported experience measures; PROMs, patient-reported outcome measures; SPKT, simultaneous pancreas-kidney transplant; QoL, quality of life; T1DM, Type 1 diabetes mellitus; T2DM, Type 2 diabetes mellitus; URO, Urology.
