# Supplementary material for: Patient Experience in Pancreas-Kidney Transplantation—A Methodological Approach Towards Innovation in an Established Program
Source: Transpl Int. 2022 Apr 14;35:10223. doi: 10.3389/ti.2022.10223 (PMC9047730; doi:10.3389/ti.2022.10223)
Supplement: Supplementary file 1 [file Table1.docx]

**Table S1.** “Living with… a pancreas-kidney transplant” patient interview script.

| **Questions** | **Domain area** |
| --- | --- |
|  | **Living with diabetes** |
| Q1 | Tell us about when you first received your diabetes diagnosis; how was this experience and how did you feel at the time? |
| Q2 | What did you know about diabetes before your diagnosis? |
| Q3 | How was your daily life with diabetes? |
| Q4 | How did diabetes change or impose limits on your life? |
| Q5 | When did you learn that you suffered from renal disease as a consequence of diabetes? |
|  | **Living with a kidney-pancreas transplant** |
| Q6 | What transplant possibilities were you told about? |
| Q7 | Who initially suggested the pancreas-kidney transplant alternative to you? |
| Q8 | What did you know about the SPKT? Where did you search for information? |
| Q9 | What expectations did you have of the SPKT? Were they met? |
| Q10 | Tell us about the experience that led to you joining the waiting list. Currently, can you be sure you received all the required patient information? Did anything unexpected happen before or after the surgery? |
| Q11 | How has the SPKT changed your life? |
| Q12 | How would you describe your daily routine and diet after receiving the transplants? |
| Q13 | How has your personal, emotional and social life changed? |
| Q14 | If you could make any changes to the whole transplantation process, what would they be? (Including initial information, joining the waiting list, post-transplant management, etc.) |

SPKT, simultaneous pancreas-kidney transplant
