# Supplementary material for: Patient Experience in Pancreas-Kidney Transplantation—A Methodological Approach Towards Innovation in an Established Program
Source: Transpl Int. 2022 Apr 14;35:10223. doi: 10.3389/ti.2022.10223 (PMC9047730; doi:10.3389/ti.2022.10223)
Supplement: Supplementary file 2 [file Table2.docx]

**Table S2.** Results of the logistics survey carried out during the COVID-19 pandemic.

| **Domain** | **Summary of patient’s answers** | **Suggestions and other notes** |
| --- | --- | --- |
| Visiting hours | They are too restricted.  Some families that live far away from the hospital prefer to wait until the afternoon/evening visiting hours. | The patient hotel could offer 24h-family visiting hours. |
| Communication with the family | Communication needs are usually covered by using a smartphone. | The patient hotel facilitates face-to-face communication and patient visits. Free wi-fi connection should be offered too. |
| Travelled distance between the hospital and home | Depending on the distance, patients may receive visits every day or may be alone if carers live too far away. | Some families prefer daily travel rather than paying for accommodation. |
| Hospital travel costs | Accommodation for friends and relatives is currently costly (up to €1,900/month).  Apart from direct travel expenses, time costs are important too. | The patient hotel may result in great savings for the families, otherwise at least some form of economic support should be offered towards travel/accommodation costs.  There are other case examples within the HCB where accommodation support is available for patient relatives, such as the Josep Carreras Foundation. |
| Boredom at the hospital | Being alone at the hospital while not perceiving any active treatments can be hard for patients. | Having free TV in the hospital room would be an asset.  The patient hotel could offer several types of activities for patients such as leisure activities, rehabilitative care and therapeutic education. |
| Level of physical activity | Moving outside the hospital room should be an option worth considering. | Having common spaces for patients at the hotel is an interesting idea. |
| Visitor comfort | It is difficult to rest on hospital visitor chairs: The seats are too small and uncomfortable, and more space is needed in general. | The patient hotel should overcome all these comfort issues. |
| Meals | In general, hospital meals are good. | Nothing to add. It is not the main issue to be addressed. |
| Attention by professionals | It is currently excellent. | Nothing else to add. |
| Medical criteria | Doctors and the rest of hospital services are trusted by patients. | Nothing else to add. |
| Hospital stays | Shorter hospital stays are preferred, but patients understand and trust medical criteria when it comes to the length of hospital stays. | The patient hotel seems to be a good solution to reducing stays and making them more comfortable while maintaining direct medical control and management. |
| Safety | The patient hotel is an attractive proposition, depending on the degree of patient dependency.  Patients also consider it a safe alternative after the first post-transplant stage. | The patient hotel should be conveniently located very close to the main hospital. |

HCB, Hospital Clínic of Barcelona
