# Supplementary material for: Patient Experience in Pancreas-Kidney Transplantation—A Methodological Approach Towards Innovation in an Established Program
Source: Transpl Int. 2022 Apr 14;35:10223. doi: 10.3389/ti.2022.10223 (PMC9047730; doi:10.3389/ti.2022.10223)
Supplement: Supplementary file 3 [file Table3.docx]

**Table S3.** Focus group script: Healthcare process & information.

| **Welcome and introduction of the objectives** | |
| --- | --- |
| **Methodology** | |
| **Introduction of the participants** | |
| **Discussion** |  |
| ***Questions*** | ***Stages*** |
|  | **Pre-transplant** |
| Q1 | How many healthcare professionals did you consult before arriving at the HCB? |
| Q2 | Did you experience any difficulty in contacting and accessing the HCB? |
| Q3 | Did you receive contradictory diagnostic information regarding your disease? |
| Q4 | How much information did you receive prior to your transplant? |
| Q5 | Were all your doubts adequately addressed? |
| Q6 | How would you describe the waiting period? |
|  | **SPKT and post-transplant** |
| Q7 | Overall, do you think you were adequately prepared for your intervention? |
| Q8 | Was the information you were provided with after the surgery sufficient and was it easy to understand? |
| Q9 | Was the information you had previously received useful at this stage? |
| Q10 | What aspects were not well informed (if any)? |
|  | **Current everyday life and improvement opportunities in the transplant unit** |
| Q11 | Did you encounter any changes at work? |
| Q12 | Did you have an understanding and supportive social environment? |
| Q13 | What needs do you feel are still not sufficiently covered? |
| Q14 | What would you improve about the healthcare service received? |
| **Identification of unmet needs and voting** | |
| **Farewell** | |

HCB, Hospital Clínic of Barcelona; SPKT, simultaneous pancreas-kidney transplant
