# Supplementary material for: Patient Experience in Pancreas-Kidney Transplantation—A Methodological Approach Towards Innovation in an Established Program
Source: Transpl Int. 2022 Apr 14;35:10223. doi: 10.3389/ti.2022.10223 (PMC9047730; doi:10.3389/ti.2022.10223)
Supplement: Supplementary file 4 [file Table4.docx]

**Table S4.** Focus group script: Nutrition and non-nutritional habits.

| **Welcome and introduction of the objectives** | |
| --- | --- |
| **Methodology** | |
| **Introduction of the participants** | |
| **Discussion** |  |
| ***Questions*** | ***Stages*** |
|  | **Before consultation with the transplant unit** |
| Q1 | Did you receive clear advice on the most appropriate diet for you? |
| Q2 | Did you receive any written instructions and/or recommendations? |
| Q3 | Were other non-nutritional habits covered too (exercise, smoking, etc.)? |
| Q4 | Were you in touch with any nutrition experts? |
| Q5 | What were the main nutritional challenges? Was it difficult to follow the recommendations? |
|  | **At the transplant unit, before the surgery** |
| Q6 | What kind of information did you receive at this point? Was it sufficient and was it easy to understand? |
| Q7 | Were other non-nutritional habits covered too (exercise, smoking, etc.)? |
| Q8 | What were the main benefits you felt from consulting with the nutritionist? |
| Q9 | What were the main nutritional challenges before the transplant? |
|  | **After the surgery** |
| Q10 | Was the information you had previously received useful at this stage? |
| Q11 | What was the essential knowledge at this stage? |
| Q12 | What are the main nutritional challenges after the transplant? |
| Q13 | To you, how important is the role of a nutritionist? |
| Q14 | Would you prefer site visits or remote consultation (telephone or videocalls)? |
| **Identification of improvement opportunities** | |
| Q15 | What would you do to enhance the transplant-related nutritional habits support? |
| **Farewell** | |
